# Type I and Type II Interferon Antagonism Strategies Used by *Paramyxoviridae*: Previous and New Discoveries, in Comparison

**DOI:** 10.3390/v14051107

**Published:** 2022-05-21

**Authors:** Giuseppe Pisanelli, Ugo Pagnini, Giuseppe Iovane, Adolfo García-Sastre

**Affiliations:** 1 Department of Microbiology, Icahn School of Medicine at Mount Sinai, New York, NY 10029, USA; giuseppe.pisanelli@mssm.edu; 2 Global Health and Emerging Pathogens Institute, Icahn School of Medicine at Mount Sinai, New York, NY 10029, USA; 3 Department of Veterinary Medicine and Animal Production, University of Naples Federico II, via Federico Delpino 1, 80137 Naples, Italy; upagnini@unina.it (U.P.); giuseppe.iovane@unina.it (G.I.); 4 Department of Medicine, Icahn School of Medicine at Mount Sinai, New York, NY 10029, USA; 5 The Tisch Cancer Institute, Icahn School of Medicine at Mount Sinai, New York, NY 10029, USA; 6 Department of Pathology, Molecular and Cell-Based Medicine Icahn School of Medicine at Mount Sinai, New York, NY 10029, USA

**Keywords:** *Paramyxoviridae* family 1, type I and type II interferon 2, *Paramyxoviridae* interferon antagonism strategies 3

## Abstract

*Paramyxoviridae* is a viral family within the order of *Mononegavirales*; they are negative single-strand RNA viruses that can cause significant diseases in both humans and animals. In order to replicate, paramyxoviruses–as any other viruses–have to bypass an important protective mechanism developed by the host’s cells: the defensive line driven by interferon. Once the viruses are recognized, the cells start the production of type I and type III interferons, which leads to the activation of hundreds of genes, many of which encode proteins with the specific function to reduce viral replication. Type II interferon is produced by active immune cells through a different signaling pathway, and activates a diverse range of genes with the same objective to block viral replication. As a result of this selective pressure, viruses have evolved different strategies to avoid the defensive function of interferons. The strategies employed by the different viral species to fight the interferon system include a number of sophisticated mechanisms. Here we analyzed the current status of the various strategies used by paramyxoviruses to subvert type I, II, and III interferon responses.

## 1. Introduction

Once entered in the cellular environment to successfully replicate, viruses must overcome the main defensive barrier triggered by the immune system–the activation of type I interferon. Since its discovery in 1957 by Isaacs and Lindenmann, this cytokine and its defensive role have been extensively studied. The main transcriptions factors involved in interferon production and signaling have been characterized in their specific functions which leads, ultimately, to the activation of hundreds IFN-stimulated genes (ISGs) whose role it is to inhibit viral replication and create the antiviral state. On the other hand, viruses have evolved a number of different strategies to evade the IFN system [1].

*Paramyxoviridae* is a family within the order of *Mononegavirales* [2]. They are enveloped single-strand negative RNA viruses and contain four subfamilies: (1) *Avulavirinae* that contain three genera: *Metaavulavirus, Paraavulavirus, and Orthoavulavirus*, (2) *Metaparamyxovirinae* that contain the genus *Synondovirus*, (3) *Orthoparamyxovirinae* that contain eight genera: *Henipavirus*, *Morbillivirus*, *Respirovirus*, *Ferlavirus*, *Aquaparamyxovirus*, *Jeilongvirus*, *Narmovirus* and *Salemvirus*, and (4) *Rubulavirinae* that contains two genera: *Pararubulavirus* and *Orthorubulavirus*. Among them are listed very important human pathogens such as the measles virus (MeV), mumps virus (MuV), and the human parainfluenza viruses (HPIV). They also include many animal pathogens like the canine distemper virus (CDV) and the two highly pathogenic zoonotic viruses Nipah (NiV) and Hendra (HeV) viruses that circulate in bats and can infect many animal species, including humans, where they are responsible for severe, often lethal, encephalitis.

To effectively replicate in cells, paramyxoviruses, like many other viral species, have developed a number of mechanisms capable of overcoming the host immune system and therefore also inhibiting type I and II interferons. They use different strategies to block IFN production and signaling pathways such as degradation, disruptions, or decreasing phosphorylation of transduction factors. Mainly, the viral proteins P, V, W, and C are responsible for most of these gimmicks. In this review, we will analyze the strategies that Paramyxovirus have developed to antagonize type I and type II interferon-induced immune responses.

## 2. *Paramyxoviridae* Genome Organization

**Figure 1:**
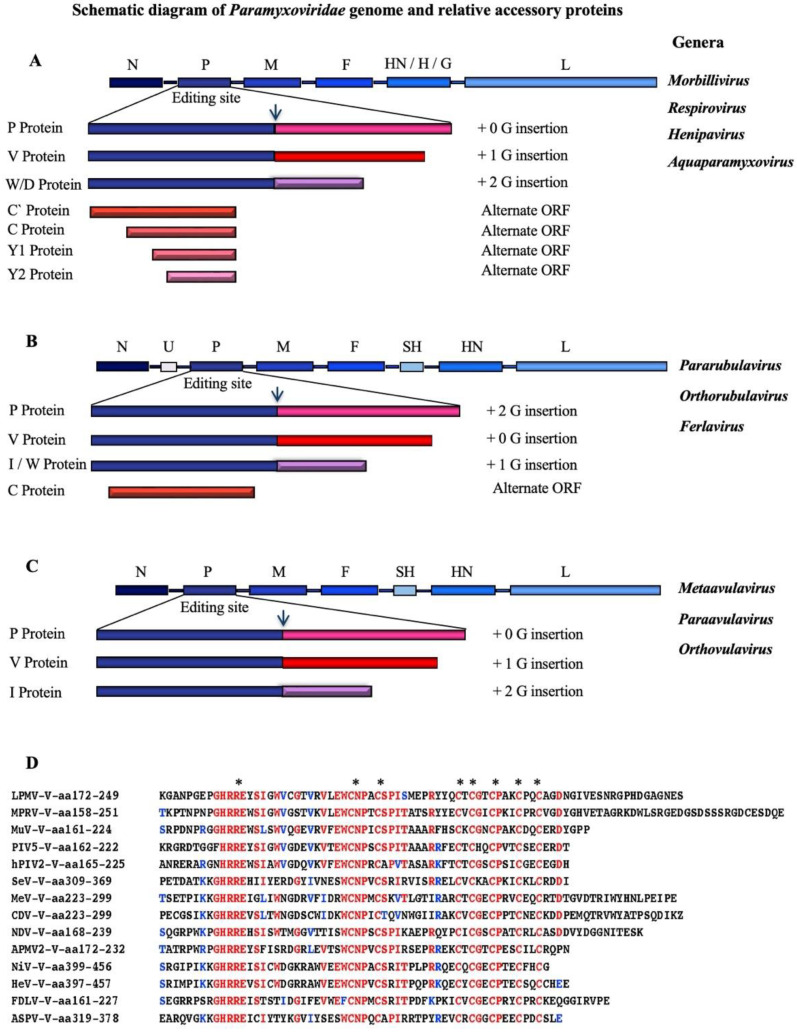
***Paramyxoviridae* genome organization, accessory proteins, and editing strategies.** (**A**) Schematic diagram of the genome organization of the genera of *Morbillivirus*, *Respirovirus*, *Henipavirus*, and *Aquaparamyxovirus*. From the left to the right: the N gene, the P gene, the M gene, the F gene, the HN gene for *Aquaparamyxovirus*, *Respirovirus* or H gene for *Morbillivirus*, or G gene for *Henipavirus* and the L gene. The P proteins of *Morbillivirus*, *Respirovirus*, *Henipavirus* and *Aquaparamyxovirus* are generated without any G nucleotide insertions at the editing site. The V proteins are generated by the insertion of one G nucleotide at the editing site. The insertion of two G nucleotides generates the W protein and D protein only for HPIV3. The C proteins are generated using alternative ORFs, starting upstream at the P ORF. SeV P gene transcribes four C proteins: (C′, C, Y1, and Y2), while HPIV3 P gene translates 3 C proteins (C′, C and Y1). (**B**) Schematic diagram of the genome organization of the genera of *Pararubulavirus*, *Orthorubulavirus*, and *Ferlavirus.* From the right to the left: N gene, U gene (present only in *Ferlavirus*), P gene, M gene, F gene, SH gene (present in *Pararubulavirus* and *Orthorubulavirus*), HN gene, and L gene. The P proteins of *Pararubulavirus* and *Ferlavirus* are produced by insertion of two G nucleotides at the editing site, while the V proteins are the result of no G insertion at the editing site. The insertion of one G at the editing site can generate I or W proteins. The C proteins are generated from an alternate ORF from a P gene. (**C**) Schematic diagram of genome organization of the genera of *Metaavulavirus, Paraavulavirus, and Orthoavulavirus.* From the right to the left: the N gene, P gene, M gene, F gene, SH gene, HN gene, and L gene. The P proteins of *Paraavulavirus* are the result of no insertion of G nucleotides at the editing site, while the V proteins are produced by one G insertion at the editing site. The insertion of two G at the editing site can generate I proteins. (**D**) Alignment of conserved cysteine-rich zinc-binding domain at the C-terminal domain of V proteins among Paramyxoviridae genera. The conserved residues are highlighted in red color and marked on top with asterisks. LPMV: La Piedad Michoacán Mexico Virus, MPRV: Mapuera virus, MuV: Mumps virus, PIV5: Parainfluenza virus 5, HPIV2: Human Parainfluenza virus 2, NDV: Newcastle disease virus, APMV2: Avian paramyxovirus 2, MeV: Measles virus, CDV: Canine distemper virus, HeV: Hendra virus, NiV: Nipah virus, SeV: Sendai virus, ASPV: Atlantic Salmon Paramyxovirus, FDLV: Fer-de-Lance virus.

The paramyxoviruses have a small genome, thus the number of viral proteins encoded is limited. They possess the N gene encoding the nucleoprotein (NP) that tightly encapsidates the viral RNA, the P gene that encodes the phosphoprotein (P), part of the polymerase complex, the M gene encoding for the matrix protein (M) important for virus morphology, assembly, and budding, the F gene that encodes the fusion protein (F), which mediates the fusion between the virus and the cell membrane, and the L gene encoding the RNA-dependent RNA polymerase (L) protein. *Aquaparamyxovirus*, *Respirovirus*, *Ferlavirus*, *Metaavulavirus*, *Paraavulavirus*, *Orthoavulavirus*, *Pararubulavirus*, and *Orthorubulavirus* possess the HN gene, which is called H gene for *Morbillivirus*, or G gene for *Henipavirus*, encoding, respectively, for HN, H, or G proteins, which all are responsible for the attachment of the virus to the cells (Figure 1A–C). These are receptor-binding proteins whose names have more recently been unified as RBP [3,4]. The *Ferlavirus* possesses a unique gene called U gene that encodes for a small protein whose function still needs to be determined. The SH gene is present in the genome of some genera of the subfamily (*Rubulavirinae* and *Avulavirinae*). This gene encodes the small hydrophobic (SH) protein that seems to function as a viroporin (Figure 1A–C) [5,6,7,8].

The P gene of most genera in the *Paramyxoviridae* family encodes for, in addition to the P protein, different non-structural proteins V, W, C, D, or I. A characteristic these proteins have in common is to have as their main function, although with different degrees, the antagonism of the interferon response.

Some of these viral proteins are encoded in the same viral gene thanks to “RNA editing” or pseudo-template addition of nucleotides, which consists in the insertion of G nucleotides by the viral RNA polymerase, in a specific position called the “editing site” in the mRNAs, which generates alternate mRNAs having a frame shift that produces an alternative ORF [9,10,11,12,13]. Every genera possesses different editing strategies, with the exception of human parainfluenza virus type 1 (HPIV1) and the cedar *Henipavirus*, which both do not have the editing site and, therefore, do not express the V or W proteins [14,15]. All the remaining viruses produce proteins from unedited mRNA, or edited with 1 or 2 G nucleotides insertions with Nipah virus reaching the highest number of G insertions (up to 11). The percentage of editing is different between the genera ranging from a lowest percentage of 31% for *Respirovirus* (SeV) to the highest of 83 % in the *Henipavirus* genus (NiV) [16].

Another mechanism that paramyxovirus uses to produce additional non-structural proteins is the use of multiple ATG initiation codons within the same mRNA. The proteins generated by these ORFs are the C proteins. Not all the genera encode for the same number of C protein. SeV, for example, encode for four C proteins from alternate ORFs in the P gene: C′, C, Y1, and Y2 proteins, the HPIV3 encodes 3 C proteins, C′, C, and Y1. NiV, but not HeV, and MeV encode for one C protein, while PIV5 does not have any C protein [17] (Figure 1A–C).

## 3. Type I and Type II Interferon

The induction of type I IFN is primed by conserved “non-self” signatures, also known as pathogen-associated molecular patterns (PAMPs), such as single-stranded RNA (ssRNA), double-stranded RNA (dsRNA), and RNA with exposed/uncapped 5′ triphosphates that are generated by infection and replication of RNA viruses recognized by PAMP-recognition-receptors (PRRs) [18,19,20,21].

Two main families of PRRs exists: cell membrane associated PRRs and cytoplasmic/nucleic PRRs. Cell membrane associated PRRs include the membrane-bound toll-like receptors (TLRs), TLR3, TLR7, TLR8, and TLR9, that are mainly expressed in immune cells and can sense extracellular PAMPs or virus-derived nucleic acids within the endosomal compartment [22,23]. The second family consists of RIG-I-like receptors (RLRs) that are ubiquitously expressed in the majority of cells and include retinoic acid-inducible gene I (RIG-I), melanoma differentiation associated gene 5 (MDA5), and the laboratory of genetics and physiology 2 (LGP2) that specifically recognize viruses that store their genetic material in form of molecules of RNA [24,25,26]. All three sensors bind double-strand RNA using their DExD/H box RNA helicase domain and C-terminal regulatory domains. LGP2 share homologous DExD/H box RNA helicase domain and C-terminal regulatory domains with RIG-I and MDA5, but lacks the N-terminal CARD domain to activate downstream signalling [27]. LGP2 displays more diverse reported roles as both a co-activator of MDA5 and a negative regulator of both RIG-I and MDA5.

Once activated, MDA5 and RIG-I proteins undergo large conformational changes leading to the interaction with the mitochondrial-associated signaling protein (MAVS also known as IPS-1, VISA, or CARDIF) through their caspase activation and recruitment domains (CARDs), which in turn leads to activation of a kinase complex composed in part of TBK-1 and IKK-ε that phosphorylates a latent cytoplasmic transcription factor known as interferon regulatory factor-3 (IRF-3). After homo-dimerization, IRF-3 translocates to the nucleus where it associates with Nuclear Factor κB (NF-κB) and activates transcription factor 2 (ATF2/c-jun) to drive IFN gene transcription [24,25,26,28].

**Figure 2:**
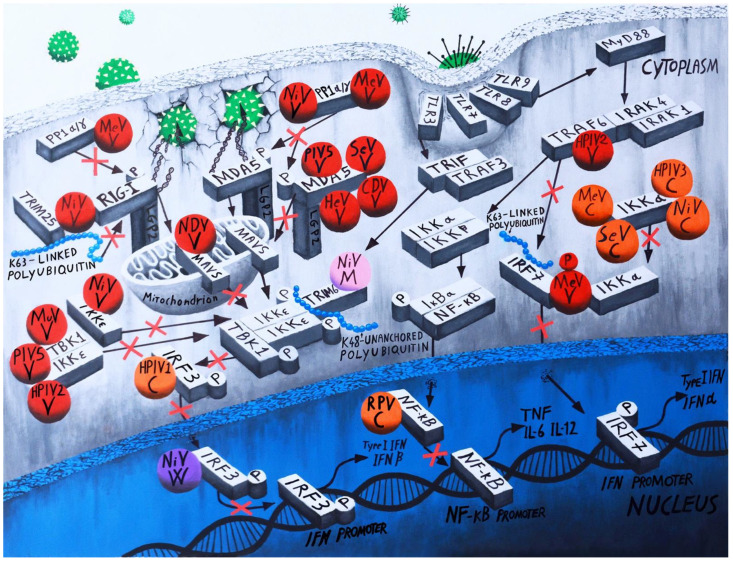
**Antagonism of interferon induction by *Paramyxoviriade***. Once activated, MDA5 and RIG-I proteins undergo large conformational changes leading to the interaction with the mitochondrial associated signaling protein (MAVS also known as IPS-, VISA, or CARDIF) through their caspase activation and recruitment domains (CARDs), which in turn leads to activation of a kinase complex composed in part of TBK-1 and IKK-ε that phosphorylates a latent cytoplasmic transcription factor interferon regulatory factor-3 (IRF-3). After homo-dimerization, IRF-3 translocates to the nucleus where it associates with nuclear factor κB (NF-κB) and activates transcription factor 2 (ATF2/c-jun) to drive IFN gene transcription. TLRs sensors activate distinct pathways, but converge with RLR, in phosphorylating IRF-3 or IRF-7, as well as nuclear factor κB (NF-κB), causing their translocation into the nucleus to activate the transcription of early type I IFNs (IFNβ and IFNα). Most of the paramyxoviruses are able to neutralize the IFN induction pathway by targeting most of the transcription factors involved in it. Most of V proteins from paramyxovirus are represented in red, interacting with IFN induction transcription factors represented in light gray, while the viruses are represented in green with yellow or black spikes. LPMV V: La Piedad Michoacán Mexico Virus V protein, MPRV V: Mapuera virus V protein, MuV V: Mumps virus V protein, PIV5 V: Parainfluenza virus 5 V protein, HPIV2-V: Human Parainfluenza virus 2 V protein, NDV-V: Newcastle disease virus V protein, MeV V: Measles virus V protein, CDV V: Canine distemper virus V protein, HeV V: Hendra virus V protein, NiV V: Nipah virus V protein, SeV V: Sendai virus V protein. Most C proteins from paramyxovirus are represented in orange, interacting with IFN induction transcription factors represented in light gray. MeV C: Measles virus C protein, NiV C: Nipah virus C protein, SeV C: Sendai virus C protein, bPIV3 C: bovine parainfluenza virus 1 C protein, HPIV1 C: human parainfluenza virus1 C protein, RPV C: Rinderpest virus C protein. NiV M: Nipah Virus M protein is represented in pink, interacting with TRIM6 IFN induction transcription factor represented in light gray. NiV W: Nipah Virus M protein is represented in violet, interacting with IRF3 activated transcription factor represented in light gray.

TLRs sensors activate distinct pathways but converge with RLR in phosphorylating IRF-3 or its close relative IRF7, as well as Nuclear Factor κB (NF-κB), causing their translocation into the nucleus to activate the transcription of early type I IFNs (IFNβ and IFNα). (Figure 2) [29,30,31,32].

**Figure 3:**
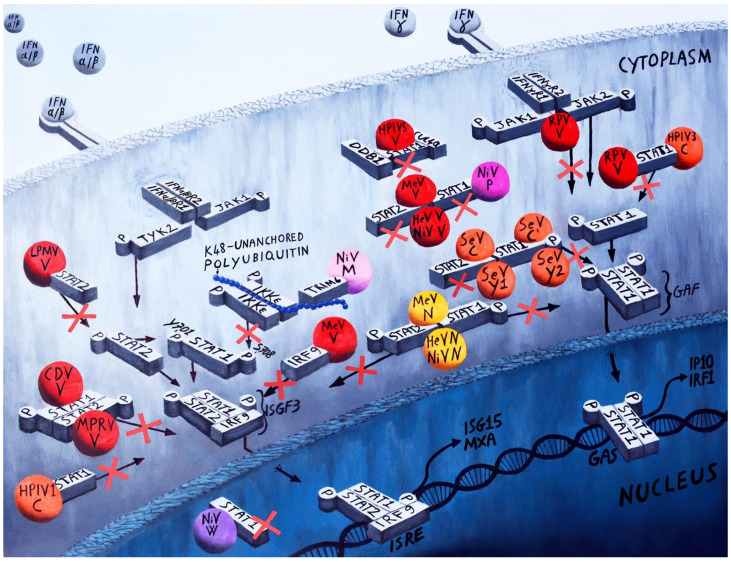
**Antagonism of type I and type II IFN signaling pathways by *Paramyxoviridae*.** Once interferon is produced, it will be released by the cells and will bind in a paracrine and autocrine manner to the interferon membrane receptors (IFNAR1 and IFNAR2). The binding with interferon receptors leads to the phosphorylation of JAK1 and TYK2. Subsequently, the activated Janus kinases activate and phosphorylate STAT2 and STAT1. Phosphorylated STAT1 and STAT2 dimerize and bind to interferon regulatory factor 9 (IRF9) to form a complex STAT1-STAT2-IRF9 called IFN-stimulated gene factor 3 (ISGF3). The ISGF3 translocates to the nucleus and binds to the DNA sequences called: IFN-stimulated response elements (ISREs) within a group of genes known as interferon-stimulated genes (ISG). This binding activates the transcriptions of more than 100 ISGs. All these genes establish an antiviral state that limits viral replication and dissemination. Type II IFN (IFN-γ) is produced by activated immune cells and leads to the production of a different subset of ISGs via a distinct signaling pathway. Type II IFN (IFNγ) binds to a different receptor complex also formed of two subunits: IFN-γR1 and IFN-γR2. Type II IFN signaling activates STAT1 by phosphorylation, which homodimerizes to form the interferon gamma factor (GAF), which translocates to the nucleus and binds to DNA at γ-activated sequence (GAS) elements within a different group of ISGs. Most of the paramyxoviruses are able to neutralize the type I and type II IFN signaling pathways by targeting most of the transcription factors involved. Most of V proteins from paramyxovirus are represented in red, interacting with type I and type II IFN signaling pathways transcription factors represented in light gray. LPMV V: La Piedad Michoacán Mexico Virus V protein, MPRV V: Mapuera virus V protein, MuV V: Mumps virus V protein, PIV5 V: Parainfluenza virus 5 V protein, MeV V: Measles virus V protein, CDV V: Canine distemper virus V protein, HeV V: Hendra virus V protein, NiV V: Nipah virus V protein, SeV V: Sendai virus V protein. Most C proteins from paramyxovirus are represented in orange, interacting with type I and type II IFN signaling transcription factors represented in light gray. NiV C: Nipah virus C protein, SeV C: Sendai virus C protein, SeV C′: Sendai virus C′ protein, SeV Y1: Sendai virus Y1 protein, SeV Y2: Sendai virus Y2 protein, bCPIV3 C: bovine parainfluenza virus 3 C protein, hCPIV1 C: human parainfluenza virus 1 C protein. Nipah Virus M protein is represented in pink, interacting with TRIM6 IFN induction transcription factor represented in light gray. N proteins are represented in yellow, forming some paramyxovirus interacting with type I and type II IFN signaling pathways transcription factors represented in light gray. HeV N: Hendra virus N protein, NiV N: Nipah Virus N protein, MeV N: Measles Virus N protein. NiV W: Nipah Virus W protein is represented in violet, interacting with STAT1 transcription factor represented in light gray. NiV P: Nipah Virus P protein is represented in magenta, interacting with STAT1 transcription factor represented in light gray.

Once interferon is produced, it is released by the cells and binds in a paracrine and autocrine manner to the interferon membrane receptors (IFNAR1 and IFNAR2). The binding with interferon receptor leads to a fast tyrosine phosphorylation of several proteins, including the α and β subunit of the IFNAR receptors. The phosphorylation of the IFN receptors activates the associated Janus kinases, Jak1, and Tyrosine kinase 2 Tyk2 [33,34]. Subsequently, the activated Janus kinases activate and phosphorylate STAT2 [35,36] on tyrosine 690 [37] and STAT1 [38,39] on tyrosine 701 [40]. Phosphorylated STAT1 and STAT2 dimerize and bind to interferon regulatory factor 9 (IRF9) to form a complex STAT1-STAT2-IRF9 called IFN-stimulated gene factor 3 (ISGF3) [41]. The ISGF3 translocates to the nucleus and binds to DNA sequences named IFN-stimulated response elements (ISREs) within a group of genes known as interferon-stimulated genes (ISG). This binding activates the transcriptions of more than 100 ISGs. The proteins encoded by these ISGs have the ability to fight viral infection using different strategies such as inhibition of viral replication, translation, viral entry, and release. All these mechanisms have the task to establish an antiviral state that limits viral replication and dissemination (Figure 3) [42,43,44,45,46,47,48,49,50,51,52].

Type II IFN (IFN-γ) is produced by activated immune cells [53,54] and leads to the production of a different subset of ISGs via a distinct signaling pathway. Type II IFN (IFN-γ) binds to different receptor complex, also formed of two subunits: IFN-γR1 and IFN-γR2 [55]. Type II IFN signaling activates STAT1 by phosphorylation, which homodimerizes to form the interferon gamma factor (GAF), which translocates to the nucleus and binds to DNA at γ-activated sequence (GAS) elements within a different group of ISGs (Figure 3).

## 4. Type I Interferon Induction by *Paramyxoviridae*

The innate immune responses against paramyxoviruses have been mainly investigated using NDV and laboratory preparations of paramyxoviruses as IFN inducers, especially vaccine strains of MeV and SeV. In fact, infection of mammalian cells with the NDV avian virus results in an uninhibited IFN response, since the V protein of NDV, which is a determinant of host range restriction, cannot interact with key mammalian cellular proteins [56,57]. Moreover, paramyxoviruses are generally weak inducers of type I IFN, but good induction emerges with virus stocks rich in defective interfering (DI) particles [58,59,60]. DI particles are sub-genomic viral RNAs generated through mistakes in virus replication [61]. DI RNAs have intact promoters but are unable to replicate on their own. DIs interfere with viral replication by sequestering the viral replication machinery [62].

It has been previously demonstrated that during infection with a Sendai virus rich in DI RNA, the DI species functioned as the main RIG-I PAMP [63].

RIG-I signaling is also regulated by PACT, which is a double-stranded RNA binding protein that binds to the C-terminal repression domain of RIG-I and function as a cellular activator to promote RIG-I-induced type I interferon production [64].

In a recent study, Ho et al., starting from the observation that the MeV Hu-191 virus is a better inducer of IFN-β than the Edmonston strain, identified a DI RNA specifically expressed by this strain. This DI-RNA was found very powerful in the IFN-β induction, and this induction was increased when RIG-I and PACT were overexpressed and absent in cells lacking in PACT or RIG-I. Specifically, DI RNA was associated with PACT showing that DI-RNA is detected by PACT and RIG-I to start a strong innate antiviral activity [60].

Paramyxovirus RNA is recognized by both endosomal toll-like receptors (TLRs), particularly TLR3 and cytoplasmic RLRs. RLRs sense viral dsRNA in the cytoplasm of infected cells [65,66,67,68,69], in particular RIG-I recognizes cytoplasmic 5′ tri-phosphorylated and uncapped viral ssRNA [70,71,72]. Several studies showed that, even though paramyxoviruses during replication encapsidate genomic and antigenomic RNAs, masking the 5′ tri-phosphorylated motifs, their RNAs mainly activate RIG-I [67,71,73,74]. Although MDA5 was for a long time considered to be superfluous for the detection of paramyxoviruses, there are several reports demonstrating that paramyxoviruses activate MDA-5 and that MDA-5 is a common target for inhibition of the Paramyxovirus V protein [75,76,77].

Our group has previously demonstrated that short 5′-triphosphate containing blunt-ended double-strand RNAs are the preferred/preferential RIG-I inducers [78], however the nature of RNA ligands of MDA5 is less clear. Recently, using crosslinking technique with next-generation sequencing, we have investigated the RNA ligands for RLR proteins from measles virus (MeV)-infected cells in vivo, demonstrating that MDA5 preferentially binds measles virus AU-rich RNA of positive polarity, whereas RIG-I additionally binds to AU-rich (−) sense RNA species originating from the MeV L gene and also from the 3′ end of the MeV genome. In vitro, these RNA molecules appear to be a poorer stimulator of the ATPase activity of MDA5, and result in more stable MDA5 filaments supporting more robust downstream signaling [79].

## 5. Role of the P, V and W Proteins in IFN Antagonism

### 5.1. Inhibition of the IFN Production by Paramyxovirus P, V, and W Proteins

Viruses of the *Paramyxoviridae* family have developed strategies to fight recognition and clearance by the immune system antagonising IFN induction [57,80,81]. Such antagonistic activity is due to one or more gene products among which the V protein is the best characterised. V proteins are characterised by a signature highly conserved cysteine-rich zinc-binding domain at the C-terminus, which is the only region of the V protein that shows significant conservation and selectively interacts with and antagonises a subset of RLRs that are required for IFN production (Figure 1D) [82,83].

The immune sensor MDA5 was first identified as a major target of paramyxovirus V proteins of several paramyxovirus (HuPV5, MuV, HuPV2, SeV, and HeV) by Andrejeva et al. in 2004, and subsequently of HPIV2, MuV, SeV, MeV, HeV, NiV, Menangle virus, Mapuera virus, Salem virus, LPMV, Tioman virus, and NDV [46,49,84]. V protein binds a specific region near residues 701–816 of the MDA5 helicase domain directly through the unique cysteine-rich binding domain, independently of the MDA5 ligand dsRNA [85,86] blocking dsRNA-MDA5 interaction to inhibit MDA5 multimerization into the active form (Figure 2) [56,86,87,88].

Analysis of porcine MDA5 and the V protein of PIV5 demonstrated that the V protein MDA5 binding unfolds the ATPase domain of MDA5, inhibiting its ATP hydrolysis activity [46] and thereby MDA5 filament formation.

MDA5 signalling activity is also regulated by a dynamic balance between phosphorylation and dephosphorylation of its CARDs by PP1α and PP1γ [89]. Davies et al., have shown that the V proteins of MeV and NiV specifically block PP1α/γ-mediated dephosphorylation of MDA5, keeping it in the phosphorylated, inactive state, inhibiting its interaction with MAVS, and preventing downstream signalling and IFN induction [90]. MeV infects primary human DC cells by inhibiting RIGI and MDA5 interferon response. The mechanism used in this case comprehends the binding of MeV with DC-SIGN in the surface of cells, which leads to the inhibition of the dephosphorylation by a formation of a molecular complex of both RIGI and MDA5, keeping them, also in this case, in a phosphorylated, inactive state, and preventing interferon induction in human DC cells (Figure 2) [91].

While the role of paramyxoviruses V proteins in antagonizing IFN by binding MDA5 and blocking its signalling is well studied [46,84], the role of V proteins in RIG-I signalling inhibition still needs clarifications. In contrast to MDA5, V proteins do not bind directly to RIG-I, nor inhibit RIG-I oligomerization or dsRNA-binding, but rather bind to another cellular helicase–the LGP2 (laboratory of genetics and physiology 2)–that in turn complexes with RIG-I to block recognition of viral RNA. Indeed, the V protein of *Parainfluenzavirus type* 5 (HuPIV5) interacts with LGP2 and inhibits induction by RIG-I ligands [49]. The V protein binds LGP2 and MDA5 through a shared interface known as the minimal V protein binding region (MVBR), located at the C-terminal domain of V that is not shared with P [49,86].

Furthermore, it has been demonstrated that the interaction of PIV5 and MuV V proteins with LGP2 specifically prevents co-activation of MDA5 signalling, not affecting the negative regulatory capacity of LGP2, but V proteins only partially antagonise RIG-I at high concentrations, and their expression has no additive effects on LGP2-mediated negative regulation [92]. Some data confirmed that both RIG-I and MDA5 induce type I IFN in cells infected with Measles Virus [93]. However, regarding the RIG I inhibition mediated by paramyxoviruses, there are discordant data in literature, and the mechanism by which paramyxoviruses inhibit RIG I remains uncertain [46,86,88,94]. Recently, we were able to demonstrate in our lab that the V protein from Nipah virus is able to interact with both RIG-I and TRIM25. The C-terminal conserved region of the V protein mediates this interaction. The interaction was also confirmed with V proteins from SeV, MeV, and HPIV5. In particular, we demonstrated the binding between V proteins, RIG-I, and TRIM25 occurs between the CARDs domain of RIG-I and the SPRY domain of TRIM25, preventing the ubiquitination of RIG-I by TRIM25, an important step in RIG-I activation [95] (Figure 2).

Other mechanisms involved in IFN production antagonism by paramyxoviruses target signalling components downstream of PRRs to prevent activation of IRF-3, as a mechanism to inhibit signalling by both RLRs and TLRs. The V protein of PIV5, MuV, and hPIV2 of the *Rubulavirinae* subfamily interacts with and inhibits IRF3/7 kinases, TRAF family member-associated, NF-kB activator, (TANK)-Binding Kinase 1 (TBK1), and Inhibitor of Kappa B Kinase epsilon (IKKε), which reside downstream of both MDA5 and RIG-I in the type I IFN induction cascade (Figure 2) [96]. Kitagawa et al. showed that the HPIV2 V protein is able to block the TLR7/9-dependent signaling, which leads to IFN-α production, by binding to TRAF6 and inhibiting TRAF6-mediated K63-linked polyubiquitination of IRF7 (Figure 2) [97]. Nevertheless, V protein of NiV has been shown to inhibit only transactivation by IKKε but not TBK1. In contrast, the NiV W protein interferes with the activated form of IRF3 in the nucleus (Figure 2) [98]. The V proteins of some Paramyxoviruses may have further functions in blocking type I IFN induction by acting as TLR7 and TLR9 antagonists. In fact, Pfaller and Conzelmann have shown that V protein of MeV down-regulate type I IFN production by TLR7 and TLR9 in plasmacytoid dendritic cells (pDCs) and conventional dendritic cells (cDCs). The V protein of MeV prevents toll-like receptor 7/9-mediated interferon induction by binding to IKKα and IRF-7, resulting in phosphorylation of V on the expense of IRF7 phosphorylation in vitro and in living cells (Figure 2) [99].

### 5.2. Inhibition of Interferon Signaling by Paramyxovirus P, V, W Proteins

Without a doubt, the main target of paramyxovirus V proteins to antagonize the IFN signaling pathway are the STATs proteins. [57,82,100,101].

Two highly pathogenic viruses, Hendra virus (HeV) and Nipah virus (NiV), isolated in Australia and in Malaysia, respectively, in the late 90s [102] showed, since the first studies, a strong inhibition of IFN signaling by targeting STATs proteins. In fact, in both viruses, classified in a new paramyxovirus genus *Henipavirus* [103], the V protein inhibits IFN signaling by forming high-molecular-weight complexes with STAT1 and STAT2 proteins and preventing STAT1 and STAT2 phosphorylation and translocation to the nucleus (Figure 3) [104,105,106]. The P, V, and W proteins have 407 amino acids in common in the N-terminal region that is responsible of the binding with STAT1 and the IFN antagonism activity of all the three proteins, with a lesser extent of IFN inhibition activity of P protein. Nipah virus V and P proteins bind STAT1 in the cytoplasm, while the W protein binds STAT1 in the nucleus (Figure 3). Interaction of HeV and NiV V proteins with STAT1 is possible through a region in their common N terminal domain of amino acid 100 to 160 [107,108]. Hagamaier et al. showed that a single amino acid change (G125E) in the STAT1/2-binding region of NiV V is critical for its ability to bind to STATs and inhibit IFN signaling [109]. Since Nipah P, V, and W proteins share the same amino terminal domain in a series of deletion mutant of P protein, Ciancanelli et al. found that the deletion of a region between amino acid residues 111 and 140 renders P incapable to bind STAT1, and thus to inhibit interferon signaling in a reporter assay, showing a consistency with the finding that a single amino acid mutation (G125E) is critical for V-STATs interaction and IFN signaling inhibition [110].

The roles of V and W proteins were studied in the contest of infection since reverse genetics became available [111]. A first study on the role of V, W, and C proteins in pathogenicity in vivo was conducted using a hamster model. Using different NiV KO for V, W, and C proteins, the authors found that V and C proteins play a major role in pathogenicity while W protein does not [112].

In a study, Satterfield et al., using a different backbone, generated a recombinant NiV (rNiVs) lacking for V and W proteins. Using ferrets as an animal model, they showed that the V protein is the principal factor of pathogenesis and lethality, demonstrating that the KO V virus is not lethal, confirming the study by Yoneda et al. that attributes the main role in NiV pathogenicity to the V protein. Most interestingly, they also found that the KO W virus modulates the inflammatory response with an effect on the disease course, with a decrease in the respiratory disease and an increase in neurological symptoms, but no alteration to lethality [113].

A second study by the same group using the same ferret model and recombinant Nipah viruses (rNiVs) lacking C and W proteins showed both W and C proteins do not seem necessary in NiV pathogenesis in the ferret model; they did not contribute to a decrease in lethality, but they both contributed to a decrease in the respiratory disease caused by NiV infection and the level of destruction of splenic germinal centers, without affecting the neuroinvasion of the virus and the neurological symptomatology [114]. Although it appears that the W and C proteins interact with transcriptions factors involved in interferon induction and signaling and inhibit the innate immune response [98,108,115,116], they are dispensable for lethality. In a similar study, the same group evaluated the most important single amino acid mutation in the common N-terminal of the NiV P/V/W proteins responsible for STAT1 binding and inhibition. They introduced a mutation in NiV that prevents STAT1 inhibition by P/V/W, and demonstrated that in ferrets, this mutation did not change lethality [117]. These results suggest some redundancy in IFN antagonism mechanisms by NiV in contributing to lethal disease.

Recently, in the genus of *Henipavirus*, a new virus, Cedar Paramyxovirus, was identified in 2009 from a flying fox colony in Cedar Grove, southeast Queensland. Since the first studies with animal models susceptible to *Henipavirus* infection such as ferrets and guinea pigs, the virus was found to be non-pathogenic. Another important characteristic is that P gene does not have V and W proteins because it is lacking the RNA editing site [15].

Lieu et al. investigated the potentiality of P protein to inhibit IFN signaling. They found that CedPV P protein is unable to bind STAT1 STAT2 proteins, inhibiting their phosphorylation and translocation to the nucleus, and thus is unable to inhibit STATs mediated IFN signaling [118].

In the *Rubulavirinae* subfamily, the HuPIV5, HuPV2, and MuV virus have a common mechanism of type I and type II IFN signaling inhibition. They employ their V proteins for STAT1 and STAT2 proteasomal mediated degradation. [119,120,121,122,123]. Parisien et al. showed that the degradation of endogenous STAT1 by HuPIV5 V protein does not occur in cells lacking STAT2 protein. In the same way, the HuPIV2 V protein does not induce endogenous STAT2 degradation in cells lacking STAT1 [122,123,124]. In order to target STATs proteins for proteasome degradation, *Rubulavirinae* V proteins, through their carboxyl-terminal domain, need the assembly of a ubiquitin ligase complex that includes DNA binding protein 1 (DDB1) and cullin4a (Cul4a) (Figure 3) [125,126,127,128,129].

A characteristic of MuV V protein is the ability to degrade through VDC (V-Dependent Degradation Complex) polyubiquitination, not only STAT1 but also STAT3, which occurs independently of STAT1 and STAT2 presence. Ultimately, MuV V protein can inhibit type I and type II interferon response and suppress cytokine and oncogene signals, showing the possibility to use Mumps Virus as an oncolytic agent. [120,123,130,131]. A recent publication indeed showed that a series of recombinant Mumps viruses possessed an oncolytic activity against tumor cell lines in vitro, and some efficacy in preliminary pilot animal tumor models [132].

The *Orthorubulavirus* LPMV is an animal pathogen that causes infertility and neurological symptoms in pigs. In a recent study conducted in our lab, we showed that the LPMV-V protein can inhibit type I IFN response, but not type II, with a mechanism that does not induce STAT1 and STAT2 proteins degradation. This contrasts with V proteins from other members of the *Rubulavirinae* subfamily. LPMV-V protein IFN evasion is similar to that of *Henipavirus* V protein with its selective binding with STAT2 protein, though not with STAT1, and with the inhibition of STATs phosphorylation and subsequent nuclear translocation (Figure 3) [133].

In the *Rubulavirinae* subfamily, Mapuera virus (MPRV) also uses a similar mechanism used by LPMV-V protein to evade IFN signaling. In fact, MPRV V protein inhibits IFN signaling by binding both STAT1 and STAT2 independently, and preventing their nuclear translocation without inducing degradation or affecting their phosphorylation (Figure 3) [134]. Two viruses in the *Rubulavirinae* subfamily are unable to inhibit IFN signaling: HuPIV 4 and TioV [135,136].

In recent studies, Young et al. and Adrianeva et al. showed that PIV2, PIV5, and mumps virus (MuV) react differently in a cellular environment with a preexisting IFN-induced antiviral state, compared with the other paramyxoviruses tested PIV3, Sendai (SeV), and canine distemper virus (CDV). All the *Rubulavirinae*, but not the other Paramyxovirus tested, were sensitive to the antiviral activity of IFIT1 because their mRNAs can be directly inhibited by IFIT1 since their mRNAs are not methylated properly [137,138].

*Morbillivirus* V proteins can use different strategies to block type I and type II interferon signaling pathway, mainly targeting multiple components of the IFN signaling pathways, like: STAT1, STAT2, Tyk2, and Jak1 [139].

The measles virus V protein inhibits type I and type II Interferon signaling response by binding with both STAT1 and STAT2, without inducing degradation or the inhibition of STATs phosphorylation, but consequently preventing their nuclear translocation in a way that resembles the mechanism used by NiV and HeV V proteins (Figure 3) [140,141,142]. Nagano et al. in a recent study found that the interaction of the C-terminal domain of V protein with STAT1 and the N-terminal region of V protein with STAT2 protein are independent of each other, and that the STAT2 binding with V protein is one order stronger than STAT1 binding with V protein. They also found a novel mechanism by which MeV V protein in addition to block STAT2/IRF9 interaction is able to disassemble the already formed interferon-stimulated gene factor by removing the STAT2-core, a central region of STAT2 that lacks the C-and the N-terminal domains, from the already formed complexes of STAT2-core/IRF9, and thus inhibit the signaling cascade [143].

Some other studies reported that MeV V protein is incapable of inhibiting type II interferon, but suppresses type I by inhibiting STAT1 and STAT2 phosphorylation in HeLa cells constitutively expressing V protein [144], or by blocking JAK1 mediated phosphorylation of STAT1 [140]. Palosaari et al. showed, in affinity purification experiments, that MeV V protein binds to the IRF9–a unique feature of MeV–probably to maximize the inactivation of the ISGF3 [141] (Figure 3). The STAT3 binding, instead, is a characteristic shared with HuPV5, and so far they are the only two paramyxovirus able to do it [141]. Canine distemper virus CDV is an important animal pathogen that causes systemic disease and encephalitis in dog. CDV V protein is capable of inhibiting type I interferon signaling by blocking STAT1 and STAT2 nuclear translocation without affecting STATs phosphorylation (Figure 3) [145]. Svitek et al., in an experimental infection carried out with ferrets infected with a wild type and a recombinant virus in which V protein is incapable to bind STAT1, showed that they developed a serious leucopenia and died within 14 days. Animals infected with recombinant viruses, in which V protein is incapable to interact with MDA5 or STAT2, or both, developed a mild disease similar to animals infected with the V-Protein-Knockout virus, showing the primary importance of interference with STAT2 and MDA5 for CDV innate immune-evasion [146].

RPV affect both type I and type II interferon signaling mediate responses by blocking the phosphorylation and nuclear translocation of STAT1 and STAT2 protein. The P and C protein were tested for type I interferon inhibition, and both showed a weaker inhibition compared to V protein [147]. The RPV/V protein possesses a strong ability to inhibit IFN γ. The probable explanation is due to the ability of the V protein of RPV to bind STAT1 and to also inhibit Jak1 phosphorylation, while V proteins MeV, CDV, and PPRV, considered in the same study, are unable to inhibit Jak1 phosphorylation but still bind STAT1 and at least partially inhibit the γ IFN signaling. The RPV V protein has two domains with distinct functions: The N-terminal domain is able to bind STAT1, whilst the C-terminal V-specific domain interacts with the IFN receptor-associated kinases Jak1 and Tyk2. Effective blockade of IFN signalling requires the intact V protein (Figure 3) [148].

The V protein of NDV is a main virulence factor. In fact, recombinant virus with a deletion for the V protein showed an attenuated growth in chicken embryos compared with a wild type strain expressing the V protein. Additionally, only the infection with wild type virus showed a degradation of STAT1 protein [149,150]. The V protein has the ability to block IFN production in chicken cells but not in human cells. In fact, exogenous expression of V during type I IFN treatment of chicken embryo fibroblasts (CEF) prevented the antiviral action of IFN [151]. A recent paper showed a new mechanism by which NDV blocks the IFN activation: the V protein of NDV degrades MAVS mitochondrial protein through the proteasome by recruiting the E3 ubiquitin ligase RNF5 to polyubiquitinate and degrade MAVS. This degradation leads to the blocking of the subsequent signal cascade, and thus the IFN production. To date, this is the first report showing MAVS degradation mediated by a paramyxovirus [152].

### 5.3. Role of Paramyxovirus C Proteins in IFN Antagonism

The V and C proteins have been demonstrated to antagonize the host interferon by several mechanisms, which were characterized for V protein in detail (see above). However, the functions of the C proteins have been less clarified. It has been demonstrated using *Morbillivirus*, *Respirovirus* and *Henipavirus* lacking the C ORF, that the C protein(s) play direct and indirect roles in limiting type I IFN induction. Strahle et al. have shown that Sendai virus mutants, lacking V or C gene expression, showed higher levels of IFN production than the wild-type virus [74], and the same scenario was described for a MeV that cannot express the C protein [153,154]. Siering et al. in a recent publication clarified an intriguing feature of the CDV with disrupted C open reading frame. They showed that a CDV lacking the C protein maintains virulence when used to infect ferrets. Interestingly, they found that the virus is able to produce, from alternative downstream start codons, truncated forms of the C proteins, despite the disruption of the first start codon. A CDV with additional abolition of these ORFs demonstrated attenuation in ferrets and in cell cultures, showing a similar phenotype with the C deficient MeV, and thus stablishing a central role of C proteins in the viral pathogenesis and virulence of both, CDV and MeV [155].

SeV virus infection lacking C, C′, Y1, and Y2 proteins resulted in a potent interferon β production, leading to IRF-3 pathway activation. By contrast, IRF3 phosphorylation and dimerization were inhibited in wild type SeV expressing C, C′, Y1, and Y2 proteins (Figure 2) [156]. In SeV infection, the C protein neutralizes RIG-I-dependent signaling and subsequent IFN production. In fact, a SeV lacking C protein is unable to prevent IFN-β activation by transfected poly (I-C) or (ppp)RNAs [74]. Moreover, infection with SeV and human parainfluenza virus type 1 HPIV1 lacking C protein leads to dsRNA accumulation, with concurrent activation of PKR [157,158].

The C proteins of SeV and HPIV3 are negative regulators of viral RNA polymerase [159,160,161,162]. Likewise, the MeV C protein also affects viral RNA synthesis [163,164], interacting with SHC binding and spindle associated 1 (SHCBP1) [165], but lacking the detectable ability to directly block type I IFN induction–rather, it acts by blocking the production of PAMPs [153]. Multiple mechanisms are essential for MeV IFN antagonism; indeed, MeV require both the MDA-5 antagonistic function of the V protein and the RNA polymerase inhibitory functions of the C protein. Furthermore, it has been shown that the C, V, and W proteins of NiV inhibit minigenome replication, suggesting that these proteins all play a role in controlling PAMP generation [166]. Significantly, it has been demonstrated that during infection of SeV rich in DI RNA, the DI species function as the most potent RIG-I PAMP [63,167].

Recently, using a recombinant SeVΔC, a mutant which lacks the SeV IFN antagonist protein C, our group found that in spite of growth conditions that normally inhibit DI genome accumulation, such as plaque purification and passaging at low multiplicity of infection, SeVΔC nevertheless contained a high amounts of DI RNAs and these molecules were again responsible for robust activation of RIG-I, stimulating IFN production [168]. Infections with wild-type SeV failed to activate RIG-I, and this is related to the lack of DI genomes in infected cells. Exogenous addition of the C protein during SeVΔC infection led to a reduction in the number of DI RNAs, further supporting the role of the C protein as a negative regulator of DI generation. Inhibition of DI RNA generation is one of the fundamental functions responsible for IFN antagonism. MeV, CDV, and HPIV 1 C proteins are also efficient DI RNA reducers. In fact, MeV CDV and HPIV 1 lacking C proteins are strong IFN inducers due to the abundant presence of DI RNA in infected cells, which activate RIG-I, MDA5, and PKR, thus leading to IFN production and viral attenuation [155,158,169]. The mechanism by which these C proteins decrease DI RNA accumulation is not completely clarified; they seem to use a different mechanism in various viral species. MeV C protein is able to bind to the ribonucleocapsid by the phosphoprotein P and to N protein [169,170]. These bindings may support the encapsidation process of the new forming RNA. In fact, lack of C protein by MeV results in premature ending of RNA synthesis. In comparisons with MeV C protein, SeV C protein binds with L protein and inhibits viral RNA synthesis [171]. In a similar way, Riderpest Virus C protein binds to L protein [172].

It is noteworthy that the C protein but not the V protein of Rinderpest virus (RPV) inhibits the induction of IFN in infected cells [173]. RPV C protein intervenes downstream of IRF-3 and NF-κB localization in the nucleus and may act like the W protein of NiV [98] (Figure 2). In a similar manner C protein of MeV interacts indirectly with IRF3 in the nucleus without affecting IRF3 dimerization, phosphorylation, and nuclear localization, and nevertheless inhibits the IFN induction [174]. Surprisingly, studies by Sanz Bernardo et al. using mutants of Peste des Petit Ruminants Virus (PPRV), a *Morbillivirus* highly related to RPV that lack expression of either of the viral accessory proteins V and C, demonstrated that PPRV V protein bound to MDA-5–and, to a lesser extent, RIG-I, and its overexpression–inhibited IFN-β induction. PPRV C protein wasn’t able to bind MDA-5 and RIG-I, but PPRV lacking C protein expression lost the ability to block both MDA-5 and RIG-I mediated IFN induction [175]. A recent paper confirmed that C protein of PPRV blocks RIG-I or MAVS mediated IFN β reporter activation [176]. Interestingly, using C protein from different genera, in particular SeV, bPIV3, MeV, and NiV C proteins, Yamaguchi et al. showed that all the C proteins bind to IKKα and inhibit IRF7 phosphorylation, and thus block toll-like receptor (TLR) 7- and TLR9-dependent IFN-α induction, which is a hallmark of activation of plasmacytoid dendritic cells (Figure 2) [115].

Finally, inhibition of activation of IRF-3 by C proteins of human parainfluenza virus 1 (HPIV1) [177] has been demonstrated, even though the exact mechanisms of action have not yet been clarified so far (Figure 2). Mutations in the C protein of the human parainfluenza virus type 3 (HPIV 3) affect viral replication and host interferon induction. In particular, infection of cells with two viruses with mutated C proteins activated the IFN regulatory transcription factor 3 (IRF-3) and subsequent increase in IFN-β mRNA levels [178]. A new study showed a novel mechanism by which C protein from SeV is able to suppress NO production in infected RAW264.7 macrophages. In particular, the SeV C protein seems to restrain the production of dsRNA, and by that precludes NF-κB activation, iNOS expression, and NO production [179].

The role of C proteins in the inhibition of the IFN signaling seems to be more important in *Respirovirus* as compared to the other genera in the *Paramyxoviridae* family. The recently discovered *Caprine respirovirus 3* (CPIV-3) possesses a V and a C protein, but only the latter is responsible for type I IFN signaling evasion by reducing STAT1 expression and phospho-STAT1 activation [180]. Some of the viruses in the genus encode for more C proteins. HPIV1, for example, does not produce the V protein, but on the other hand is able to produce three proteins, C′ C, and Y1, from the P gene using alternate ORFs [14,181]. The role of a V protein as an IFN antagonist in HPIV1 seems to be efficiently taken over by the C protein. In fact, this protein is able to inhibit type I IFN signaling by binding and retaining STAT1 in aggregates localized around the nucleus, preventing STAT1 translocation to the nucleus, and reducing STATs phosphorylation (Figure 3) [182]. The HPIV3 C proteins efficiently suppress type I and type II interferon response by inhibition of STAT1 phosphorylation, and consequentially the GAS and ISGF3 complexes formation (Figure 3) [183]. The same group showed that approximately 50% of the N-terminal region of the C-protein is responsible for the IFN signaling inhibition [184]. Interestingly, a recent study shows that HPIV3 blocks antiviral mediators downstream of the interferon lambda receptor by reducing STAT1 phosphorylation [185].

SeV P gene is able to transcribe at least seven proteins: from different start codons in the P open reading frame (ORF) are produced four C proteins (C′, C, Y1, and Y2), while the V and W proteins are produced from an editing site with the insertion of one or two G nucleotides, respectively, in a specific position of the mRNA during transcription [17]. The SeV C protein has a primary role in the inhibition of the signaling interferon response. Different groups have showed that using different SeV mutants lacking all the four C proteins are unable to block both the type I and type II interferon antiviral mediated responses [186,187]. The molecular mechanism appears to be related to the characteristic of all four C proteins to bind STAT1 and its tyrosine phosphorylated form (pY-Stat1) to form an aberrant High Molecular Weight Complex (HMWCs). This interaction prevents pY-Stat1 from binding to GAS elements and blocks its translocation to the nucleolus, thus inhibiting the activation of ISG. In particular, the C-terminal of the Sendai virus C protein is necessary to prevent the GAF from binding to a gamma-activated sequence site. Only the larger form of C proteins also induces STAT1 mono-ubiquitination and degradation. Moreover, the C protein inhibit STAT2 phosphorylation through the previous STAT1 interaction (Figure 3). [188,189,190,191,192]. Recently, the crystal structure of the interaction between STAT1 N-terminal domain and the C-terminal half of the C protein of Sendai Virus has been shown at a resolution of 2.0 A. From the structural analysis and experiments, the authors propose a model in which two C proteins bind the parallel form of STAT1 dimers and stabilized them with the consequential reduction in the phosphorylation at Tyr (701), which leads to the accumulation of P-STAT1 that together with C protein forms a high-molecular-weight complex resulting in the inhibition of type II interferon signalling [193].

From the analyzed data, it is clear that C proteins, despite not being present in all the paramyxovirus genera (they are produced in approximately one third (26 on 63) of the genome sequence of paramyxovirus species sequenced so far [194]), possess an important role in antagonizing both IFN induction and signaling pathways. The results of studies conducted in vitro with viruses deficient of C proteins are consistent with in vivo studies using animal models or natural hosts. The *Morbillivirus*, *Henipavirus*, and *Respirovirus* lacking C protein expression showed a low pathogenesis in vivo, confirming their role as virulence factors [114,194,195,196,197,198,199,200,201].

### 5.4. Role of Structural Proteins in IFN Antagonism: M and N Proteins

The M protein is one of the six structural proteins encoded by paramyxovirus. Its main function is to drive viral assembly and budding by connecting the F and/or H viral proteins and the RNPs. Up to a few years ago, the possible role played by the Paramyxovirus M protein as antagonist of the interferon had never been considered. Recently, two papers were published explaining two different mechanisms by which this protein from two different viruses evades the interferon response. In the first paper, the authors showed that NiV matrix protein (NiV-M) inhibits type I IFN induction. In our previous study, we showed that the E3-ubiquitin ligase TRIM6 activates the formation of K48-linked polyubiquitin chains and IKKε auto phosphorylation, which consequentially phosphorylates IRF3 for type I IFN mediate response [43]. This inhibition is possible because NiV- M protein interacts with TRIM6 and causes its degradation, thus blocking the subsequent signaling cascade. The data were confirmed in vivo using a wild type NiV in which they found reduction in IFN and degradation of TRIM6. In contrast, a virus lacking the M protein was deficient in these functions. The degradation of TRIM6 by NiV M protein also affects the Type I IFN signaling cascade due to the role played by IKKε in type I IFN signaling pathway in leading the expression of a subset of IKKε dependent ISGs through the IKKε specific phosphorylation site (S708) on STAT1 [202] (Figure 2 and Figure 3). The mechanism of degradation of TRIM6 by NiV-M still needs to be clarified, but a novel mechanism and a new protein employed by paramyxovirus to counteract the IFN system has been revealed by this study [203]. The role of M protein as IFN antagonist was confirmed a year later from another group, this time using parainfluenza virus type 3 (HPIV3). In particular, the authors have demonstrated that human HPIV3 induce mitophagy and that M protein plays a key role in this process. Interestingly, the authors have demonstrated that M protein of HPIV3 induces mitophagy by bridging autophagosomes and mitochondria via interactions with TUFM and LC3, simultaneously inhibiting the type I IFN response [204].

The N gene encodes the N or NP protein. The function of N protein is to form a coiled nucleocapsid structure around the viral RNA called ribonucleoprotein complex (RNP), which associates with the viral RNA-dependent RNA polymerase complexes, constituted by the P phosphoprotein and L polymerase protein. The role of the P gene and its products as IFN antagonists has been well establish in the *Paramyxoviridae* family. Since P and N proteins closely interact during replication, some researchers have explored the possibility of N protein acting as an IFN antagonist. In a first study, Takayama et al. found that MeV N protein blocks type I and type II interferon signaling responses. They showed that MeV-N blocks the nuclear import of activated STATs without affecting STAT1 and STAT2 phosphorylation or inducing their degradation, with a consequent reduction in IFNs mediated signaling responses. Interestingly, the suppression of IFNs signaling responses by the N protein was observed to also be affected by CDV and RPV N proteins, showing in both cases a stronger type I and type II interferon inhibition compare to MeV N protein [205]. In a recent study, the same group highlighted a new role of the nucleoprotein (N) of *Henipaviruses* in preventing the host IFN signaling pathway. In fact, both NiV and HeV N proteins can suppress both type-I and type-II IFN responses in reporter assays. The mechanism used by N protein consists in the reduction in the formation of functional molecular complexes of STATs, without affecting STATs phosphorylation or promoting their degradation. NiV N prevented the nuclear transport of STAT1 and STAT2 and consequent reduction in ISGs (Figure 3) [206].

## 6. Conclusions

The intention of this paper was to review the literature available to date on the immune-evasion mechanisms utilized by the *Paramyxoviridae* family to counteract type I and type II interferon response. From the papers analyzed, we can certainly note that paramyxovirus have developed different abilities to successfully avoid the interferon innate immuno-response and to replicate efficiently in their host.

To overcome the interferon’s activity, paramyxoviruses target most of the factors involved in the interferon induction and signaling pathways. In order to do so, despite their small genome, they used different mechanisms (RNA editing and alternate ORFs) to produce a variety of proteins from a P gene, with the main function of counteracting the IFN response. In general, we can immediately remark that the V protein plays a major role in the inhibition of IFN induction. In almost all the viral genera examined, the V proteins’ main target of the IFN induction are RLRs receptors. Crystallographic study showed that paramyxovirus V proteins through the highly conserved C-terminal domain (CTD) bind MDA5, which seems to have a major role in the paramyxovirus recognition. By this conserved mechanism, V proteins are able to physically destroy the MDA5 architecture, preventing RNA binding to MDA5 and thus inhibiting IFN activation. V proteins also bind LGP2, and a recent publication showed that they bind RIG-I as well. RIG-I inhibition requires two specific interactions of V protein: one to the RIG-I CARD domain, and the other with TRIM25 SPRY domain, in this way preventing TRIM25 mediated ubiquitination of RIG-I interrupting the downstream RIG-I mediated signaling.

The discoveries of new host factors that regulate interferon activation have facilitated additional discoveries in how viruses have developed in parallel strategies to subvert these factors. This is true in the case of the recent discovery of de-phosphorylation and activation of RIG-I and MDA5 by PP1α/γ. MeV and NiV keep RIG-I and MDA5 in a phosphorylation inactive status by blocking PP1α/γ. The discovery of the role played by TRIM6 in the IFN induction and signaling pathways facilitated the identification of the M protein from NiV as responsible for the degradation of TRIM6, and thus the inhibition of the interferon activation.

A broad range of studies was conducted to clarify the mechanisms by which paramyxovirus suppress IFN signaling response. As with IFN induction, the principal contribution in the IFN signaling pathway inhibition is still a prerogative of the V proteins. In almost all the viral genera examined, the V protein inhibits type I and type II interferon signaling by targeting STATs proteins. Interestingly, each virus targets STATs proteins using different strategies. Some promote their degradation, others block their translocation to the nucleus, whilst some others prevent their phosphorylation. The unique cysteine-rich C-terminal zinc-binding domain of V protein is the common denominator essential to this function.

In some genera, the role possessed by the V proteins is delegated to the C proteins, despite being very divergent in a.a. composition and lacking the unique cysteine rich C-terminal zinc-binding domain present in V proteins. These proteins have a major involvement, especially for *Respirovirus*, *Morbillivirus*, and *Henipavirus*. The studies of the C proteins as IFN antagonists are numerous and show the multifunctional role of the C proteins in targeting both the IFN induction and signaling response.

Moreover, C proteins are efficient reducers of DI RNA, thus decreasing DI RNA IFN induction mediated by RIG-I, MDA5, and PKR activation.

An important finding in recent years that needs to be underlined is the fact that paramyxoviruses are able to evade the IFN response using the structural proteins M and N. This finding is even more interesting because it is not genus-specific, but is conserved through different genera. This is the case of M proteins from NiV and HPIV3 that are able to block the IFN response using different mechanisms, or of the N proteins from HeV and NiV in *Henipavirus* genus and the MeV, CDV, and RPV in the *Morbillivirus* genus that target the STATs proteins to block the IFN signaling response.

One of the main concerns in IFN antagonism paramyxovirus studies was the lack of in vivo studies, since most of the studies were carried out with infected cell lines and/or using transfection experiments. However, in recent years and from the data reviewed above, many studies used in vivo animal models to confirm and validate the data obtained in vitro. This is the case, for example, of the ferret animal model, where ferrets were infected with the recombinant Nipah virus lacking the V, W, C genes, or, in a similar way the data obtained with *Morbillivirus* and *Respirovirus* lacking V and C genes, used in different animal models to confirm the role in vivo of the respectively encoded proteins.

In conclusion, the body of literature analyzed here highlights the wide range of mechanisms employed by paramyxovirus to counteract IFN system defense. The new and previous discoveries, discussed above, generate new ideas to develop future vaccines using the different attenuated viruses generated to date or pharmacological interventions, taking into consideration the detailed molecular mechanisms investigated so far that underline the IFN inhibition.
